# Distinct liquid-liquid phase separation properties of end-binding proteins EB1 and EB3

**DOI:** 10.1016/j.jbc.2025.110849

**Published:** 2025-10-22

**Authors:** Solomiia Boyko, Qiuye Li, Krystyna Surewicz, Witold K. Surewicz

**Affiliations:** Department of Physiology and Biophysics, Case Western Reserve University, Cleveland, Ohio, USA

**Keywords:** EB1, EB3, liquid-liquid phase separation, microtubules, tubulin

## Abstract

End-binding proteins (EBs) are central components of the network of microtubule-plus-end-tracking proteins (+TIPs) that modulate microtubule dynamics. Recent studies have shown that EBs undergo liquid–liquid phase separation (LLPS), and it was proposed that the resulting condensates could play a major role in the recruitment of other + TIPs as well as in polymerization of tubulin. Here, we performed detailed studies of LLPS properties of two major members of the EB family in mammalian cells, EB1 and EB3. Surprisingly, we found that, despite 67% sequence identity, EB3 has a significantly higher LLPS propensity than EB1, both *in vitro* and in cells. This difference is due to combined contributions from multiple protein regions, with histidine residues in the N-terminal domain playing a particularly important role. Furthermore, EB1 and EB3 condensates were found to differ in their material properties, with EB3 droplets being much less dynamic than the EB1 counterparts. Importantly, EB3 droplets had higher capacity to recruit tubulin and nucleate its polymerization. The differences with regard to the impact of condensation on tubulin polymerization were especially striking in the presence of another + TIP-associated protein, CLIP-170, in which case higher tubulin polymerization capacity was observed for EB3/CLIP-170 droplets than the EB1/CLIP-170 counterparts, and this difference was attributed to distinct material properties of the two droplet types. These findings suggest that different, EB-dependent + TIP body types may exist in cells, contributing to functional specialization of microtubules.

Microtubules are cytoskeletal filaments that play a crucial role in intracellular transport, cell shape maintenance, polarity, and cell division. They form through the polymerization of αβ-tubulin heterodimers, a process regulated by GTP hydrolysis. Microtubules are polarized, with minus ends that expose α-tubulin and plus ends that expose β-tubulin (1). The ends of microtubules undergo continuous cycles of polymerization (growth) and depolymerization (shrinkage), with occasional pauses, a phenomenon known as “dynamic instability” (2). This process is tightly regulated by various microtubule-associated proteins (MAPs) (3). Among these, microtubule-plus-end-tracking proteins (+TIPs) are a diverse group of regulators that accumulate at the growing plus ends of microtubules and are uniquely positioned to influence their behavior (4, 5, 6).

EBs are key components of +TIP networks (4, 5, 6, 7); they bind to growing microtubule ends and play a critical role in the recruitment of other partners. Mammalian cells express three distinct EBs (EB1, EB2, and EB3) that are encoded by separate genes, while yeasts have only one EB: Bim1 in *Saccharomyces cerevisiae* and Mal3 in *Schizosaccharomyces pombe* (5). The structure of EBs includes an amino-terminal calponin homology (CH) domain, followed by an intrinsically disordered region (IDR), a coiled-coil domain, a four-helix bundle, and a disordered tail, which ends with a carboxy-terminal EEY/F motif. The four-helix bundle and part of the tail region together form the EB homology domain (5, 7). The coiled-coil domain mediates the homo- or heterodimerization of EB monomers (8, 9). The C-terminal region contains binding sites for various + TIP partners. While the CH domain and IDR are sufficient for recognizing and tracking growing microtubule ends, the interaction between the CH-IDR domains and the charged C-terminal region is required to fine-tune the specificity of EBs for microtubule tips (10).

An important recent development was the discovery that at least some MAPs can undergo liquid-liquid phase separation (LLPS), a process in which proteins "demix" from solution, forming liquid droplets (11, 12, 13, 14). The capacity of these proteins to form condensates has led to the suggestions that LLPS may be involved in many aspects of microtubule biology, including microtubule nucleation, structural organization, and microtubule-based transport (11, 12, 13, 14). However, many aspects of the postulate role of LLPS in microtubule biology remain poorly understood and are controversial.

In particular, LLPS was found for the members of the +TIP network (11, 15, 16, 17, 18, 19, 20), and +TIPs condensates at the growing end of microtubules in mammalian cells (or both growing and shrinking ends in yeast) were recently dubbed “+TIP bodies” (19). Initial observations in this regard were made for EB1, which was reported to undergo LLPS at low micromolar (or even submicromolar) concentrations under physiological buffer conditions in the absence of any crowding agents (15). It was also concluded that this process is driven by electrostatic interactions between different protein domains, with Lys and Arg residues in the IDR playing a key role (15). LLPS was also observed in a subsequent study for EB3 (16), and it was suggested that condensates formed by EBs may not only serve as hubs for recruiting other + TIPs to microtubule ends, but also help concentrate tubulin at the microtubule tip, thereby facilitating polymerization process (15, 16). Furthermore, EB1 LLPS has been shown to play a critical role in cell division, particularly in chromosomal segregation (15, 21, 22).

While these data suggest an intriguing possibility that LLPS of +TIPs may be important in microtubule biology, many aspects of condensation of these proteins remain unexplored. In this study, we focused specifically on comparing the LLPS behavior of two members of the EB family, EB1 and EB3. To our surprise, we found that, despite high sequence identity (67%) (7), there are large differences in the phase separation propensity of these proteins as well as physicochemical properties of the resulting condensates. Our study also revealed that, in comparison to EB1, EB3 droplets have higher capacity to recruit tubulin as well as to nucleate its polymerization in the presence of CLIP-170.

## Results

### His-tag significantly affects the propensity of EB1 to undergo LLPS

**Figure 1:**
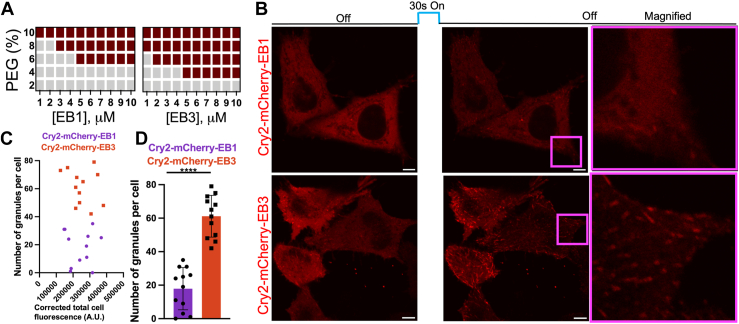
**EB1 and EB3 have different capacities for LLPS *in vitro* and *in cells*.***A*, protein concentration *versus* polyethylene glycol concentration phase diagrams for EB1 and EB3. *Gray* and *brownboxes* indicate the absence and presence of phase separation, respectively. *B*, representative fluorescence images of *blue lig*ht-activated assembly of Cry2-mCherry-EB1 (*top*) and Cry2-mCherry-EB3 (*bottom*) in HeLa cells expressing similar levels of these proteins. *Right panels* show magnified images of *pink* squares in *middle* panels. *C*, quantification of granules formed after *blue-light* stimulation in cells expressing different levels of Cry2-mCherry-EB1 and Cry2-mCherry-EB3. Corrected total cell fluorescence of mCherry was used a measure of Cry2-mCherry-EB1 and Cry2-mCherry-EB3 expression levels (see Experimental procedures section for details). *D*, cumulative number of granules in cells expressing Cry2-mCherry-EB1 and Cry2-mCherry-EB3 at the levels corresponding to mCherry fluorescence intensity in the 130,000 to 380,000 A U. range. *Error bars* represent SD (n = 12 individual cells). Statistical significance was assessed using unpaired two-tailed Student’s *t* test. ∗∗∗∗*p* < 0.0001.

It was recently reported that bacterially expressed full-length EB1 undergoes LLPS at submicromolar concentrations even in absence of any crowding agents (*e.g.*, at 0.5 μM concentration in a buffer containing 100 mM KCl) (15). Our attempts to reproduce these results have not been successful, even when the protein concentration was increased to 10 μM (Fig. 1*A*). We found, however, that EB1 at low (submicromolar) concentrations forms liquid droplets when crowding agents such as polyethylene glycol (PEG) are present in the buffer containing nearly physiological concentrations of salt (Fig. 1*A*).

This major discrepancy between the previous and present findings is not due to the use of different buffers (PIPES, pH 6.8 in the previous study *versus* Hepes, pH 7.4 in most of our experiments), as repetition of these experiments under exactly same buffer conditions as used previously also failed to reproduce the findings regarding robust LLPS of EB1 in the absence of molecular crowders (Fig. S1, *A* and *B*). In an attempt to clarify this issue, we noted that the previous study (15) was performed using EB1 fused to GFP and a His-tag. By contrast, our experiments were done with the protein without any tags (the His tag linked to the protein *via* thrombin cleavage site was removed after purification by treatment with thrombin). To test the impact of these tags on EB1 LLPS, we performed experiments using three proteins: EB1 containing thrombin cleavage site and the His tag, EB1 contains the N-terminal His tag only, and EB1 alone (in which His tag was removed by thrombin cleavage). Remarkable, these additional residues were found to have a major effect, with His tag greatly increasing EB1’s propensity for LLPS (and the sequence containing both the His tag and the thrombin cleavage site having even a more dramatic effect) (Fig. S1, *C* and *D*). Although we did not directly assess the LLPS capacity of EB1 containing both GFP and the His tag (EB1-GFP-6xHis) used in the previous study (15), our results clearly indicate that the presence of the His tag alone greatly increases EB1’s LLPS propensity. In this context, it should be noted that previous studies have shown that the His tag can also affect the interaction of EB1 with microtubules, significantly increasing its affinity to microtubules (23). Therefore, all our experiments described below were performed with the tag-free proteins.

### EB1 and EB3 have different LLPS properties

We next compared the LLPS capacity of EB1 and EB3. To our surprise, we found that, despite their high structural similarity (67% sequence identity) (Fig. S2), the two proteins have different properties in this regard, with EB3 being more prone to undergo condensation. The latter protein forms droplets at lower PEG concentration (Fig. 1*A*), and its saturation concentration [*i.e.*, the concentration above which the system starts to phase separate) under similar buffer conditions is substantially lower (*e.g.*, in the presence of 8% PEG, c_sat_ for EB3 and EB1 is 0.8 and 2 μM) (Figs. 1*A* and S3). The observed difference in LLPS propensity between EB1 and EB3 was confirmed using fluorescence microscopy. At the same protein concentration, EB3 droplets were found to cover approximately twice larger surface area than EB1 droplets (Fig. S4), further indicating higher LLPS capacity of EB3. Furthermore, control experiments using labeled and unlabeled proteins demonstrated that covalent attachment of AlexaFluor488 does not affect the capacity of either EB1 or EB3 to form droplets (Fig. S4).

To validate these findings in a cellular context, we used the optoDroplet system in which LLPS-prone protein of interest is fused to mCherry (for fluorescence detection) and Cry2, a light-sensitive protein that self-associates upon exposure to blue light (24). Blue light-induced transient oligomerization of Cry2 results in local increase in concentration of the LLPS-prone protein above its c_sat_, leading to its condensation. In our experiments, we used HeLa cells that were transiently transfected with the Cry2-mCherry-EB1 or Cry2-mCherry-EB3 plasmids and mCherry fluorescence intensity was used as a measure of expression of individual proteins Analysis of a population of cells with a range of modest expression levels of Cry2-mCherry-EB1 or Cry2-mCherry-EB3 (that show no granules without blue light stimulation) revealed that, upon blue light stimulation, substantially larger number of condensates (granules) were formed in EB3-expressing cells compared to EB1-expressing cells at equivalent protein expression levels (Fig. 1, *B*–*D*). As expected for LLPS, these condensates were highly dynamic, disassembling within 10 min after turning off the light (Fig. S5*A*). No condensates were observed when vector carrying Cry2 fused to mCherry was used as a control (Fig. S5*B*). In control experiments we verified that this observed difference in granule number in Cry2-mCherry-EB1 or Cry2-mCherry-EB3 expressing cells is not due to degradation of the fusion proteins, as both Cry2-mCherry-EB1 and Cry2-mCherry-EB3 remained intact (Fig. S5*C*). It should also be noted that in a small population of cells that expressed Cry2-mCherry-EB1 or Cry2-mCherry-EB3 at high level, both proteins formed granules even in the absence of blue light stimulation, indicating that fusion of Cry2-mCherry to EB1 or EB3 does not interfere with condensate formation (Fig. S6). However, reliable quantitative analysis of granules in this group was not practical due to a small number of cells in this population. Overall, these results indicate that EB3 has substantially higher capacity for LLPS than EB1, both in the test tube and in cells.

**Figure 2:**
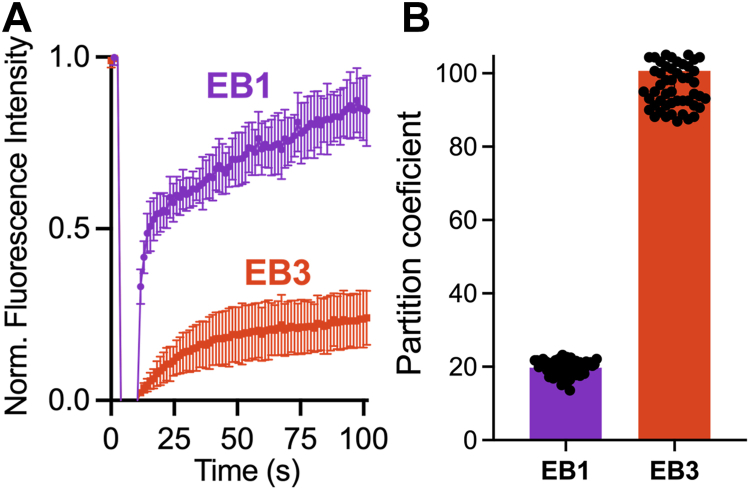
**Distinct material properties of EB1 and EB3 droplets.***A*, representative fluorescence recovery after photobleaching (FRAP) traces for droplets prepared from EB1 and EB3. The concentration of proteins in these experiments was 30 μM. Each trace represents the average of measurements for 6 to 9 droplets; *error bars* represent SD. *B*, partition coefficients between the condensed and dilute phases for EB1 and EB3. Data represent averages for least 61 droplets of each protein variant. *Error bars* represent SD. FRAP, fluorescence recovery after photobleaching.

Material properties of the droplets formed *in vitro* by EB1 and EB3 were assessed using fluorescence recovery after photobleaching (FRAP) experiments. These measurements revealed that close to 100% of fluorescence signal was recovered after photobleaching of EB1 droplets, indicating high mobility of essentially all protein molecules within these droplets (Fig. 2*A*). By contrast, the mobile fraction of protein within EB3 droplets was much smaller, at the level of approximately 25% (Fig. 2*A*). Overall, these data indicate a significantly less dynamic (more viscous) nature of EB3 condensates than that of the EB1 counterparts. One possible reason for this large viscosity difference could be higher protein concentration within EB3 droplets. To test this hypothesis, we determined for each protein the partition coefficient between the condensed and dilute phases. This coefficient was found to be five times higher for EB3 than EB1 (Fig. 2*B*), clearly indicating higher relative protein concentration within EB3 droplets as compared to that within EB1 counterparts. Thus, EB3 condensates appear to be more viscous due to higher protein concentration within them. Altogether, these data indicate that, despite similarities in the primary structure, EB1 and EB3 not only differ significantly with respect to their LLPS propensity but also form droplets with distinct material properties.

### Residues across different protein domains are responsible for distinct LLPS behavior of EB1 and EB3

Next, we aimed to determine which protein domains, and specific residues within them, are responsible for substantially higher LLPS propensity of EB3 compared to EB1. Previous studies with EB1 concluded that LLPS of the latter protein is driven by electrostatic interactions between multiple domains, with Lys and Arg residues in the IDR playing a key role in this process (15). However, sequence comparison (Fig. S2) shows that both proteins contain the same total number of Lys and Arg residues (22 and 9, respectively) and similar number of these residues within IDRs (4 Arg, 5 Lys and 5 Arg, 5 Lys in EB1 and EB3, respectively), suggesting that other amino acids are responsible for the differences in LLPS propensity of EB1 and EB3.

**Figure 3:**
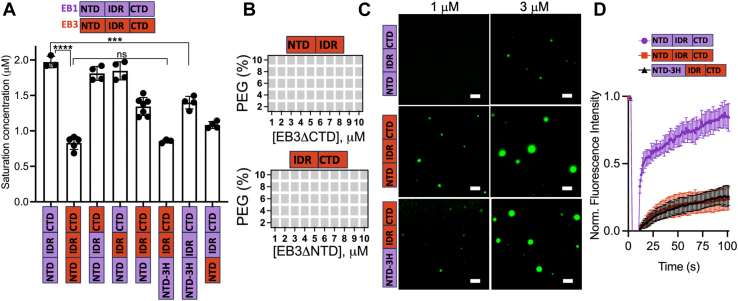
**Multiple residues across protein domains are responsible for differences in LLPS propensity of EB1 and EB3.***A*, saturation concentrations for EB1, EB3, and the hybrid variants. Error bars represent SD (n = 3–7 technical replicates). Different protein variants examined are indicated at the *bottom* using the color-coded representation of the domain structure of EB1 (*purple*) and EB3 (*red*). Statistical significance was assessed using unpaired two-tailed Student’s *t* test. ∗∗∗*p* < 0.001, ∗∗∗∗*p* < 0.0001, ns - nonsignificant (*p* > 0.05). *B*, protein concentration *versus* polyethylene glycol concentration phase diagrams for ΔNTD and ΔCTD variants of EB3. *Gray boxes* indicate the absence of phase separation. *C*, representative fluorescence microscopy images of EB1, EB3, and the EB1(NTD3H)-EB3(LCD-CTD) hybrid protein. Protein concentrations are marked at the *top* of the panels. The proteins were labeled with Alexa Fluor 488 (*green*), and the ratio of labeled to unlabeled protein was 1:10 in each case. The images were obtained ∼10 min after sample preparation; The scale bar represent correspond to 2 μm. *D*, representative FRAP traces for droplets prepared from EB1, EB3, and the EB1(NTD3H)-EB3(LCD-CTD) hybrid protein. The concentration of proteins in these experiments was 30 μM. Each trace represents the average of measurements for 6 to 9 droplets; *error bars* represent SD. FRAP, fluorescence recovery after photobleaching.

Given the important role of IDR in LLPS of EB1 (15), we first explored the possibility that the differences in this region’s primary structure (Fig. S2) are responsible for higher LLPS propensity of EB3 than that of EB1. To this end, we created a hybrid protein, EB1 N-terminal domain (NTD)-EB3(IDR)-EB1C-terminal domain (CTD), in which the IDR in EB1 was replaced with that of EB3 and tested the LLPS capacity of this protein. To our surprise, the saturation concentration of the hybrid protein was very similar to that of EB1 (Fig. 3*A*), indicating that differences in IDRs alone are not sufficient to account for different LLPS propensity of EB1 and EB3.

Next, we focused on the CTD, which has a longer predicted disordered sequence in EB3 than in EB1 (Fig. S7*A*). Akin to IDR (15), this domain appears to be also essential for droplets formation, as its deletion in EB3 resulted in the abrogation of LLPS even in the presence of high concentrations of the crowding agent (Fig. 3*B*). The importance of this domain is likely related to its overall negative charge (Fig. S7*B*), the removal of which would eliminate electrostatic interactions with NTD and IDR. To explore the specific role of CTD in distinct LLPS properties of EB1 and EB3, we generated another hybrid protein, EB1(NTD-IDR)-EB3 (C-terminal domain), in which the NTD and IDR of EB1 were fused with the CTD of EB3. Unexpectedly, swapping of the latter domain had negligible effect on LLPS propensity of EB1, as indicated by very similar saturation concentrations of WT EB1 and the hybrid protein (Fig. 3*A*). Thus, akin to the observations for IDR, differences in the primary structure of CTD alone do not account for distinct LLPS properties of EB1 and EB3. However, when both IDR and CTD in EB1 were replaced with the respective EB3 domains, we found that the resulting protein had enhanced LLPS capacity as compared to the WT EB1 (c_sat_ of 1.5 and 2.0 μM, respectively), even though still not as high as that of EB3 (c_sat_ of 0.8 μM) (Fig. 3*A*).

While these observations suggest that IDR and CTD may act in concert, synergistically contributing to the LLPS of EBs, they also point to a potentially critical role of the NTD in this process. Indeed, deletion of this domain in EB3 resulted in the loss of protein capacity to phase separate (Fig. 3*B*). Given our findings regarding a major role of the His tag in driving LLPS of His-tagged EB1 (see above), we were puzzled that NTD in EB3 contains three His residues (H29, H57, H73), which in EB1 are replaced by other amino acids (Fig. S2). This, together with structural data showing that H29, H57, H73 in EB3 are exposed on the protein surface (25), led us to the hypothesis that these His residues may play an important role in phase separation, contributing to higher LLPS propensity of EB3 as compared to EB1. To test this possibility, we substituted amino acids at positions 29, 57 and 73 in EB1 with His (Q29H, A57H, Q73H) and fused this modified EB1 NTD (3HNTD) with EB3’s IDR and CTD. Remarkably, the resulting protein was characterized by substantially increased LLPS capacity, with a saturation concentration essentially identical to that of EB3 (Fig. 3 *A* and *C*). To further confirm the LLPS-promoting role of these His residues, we introduced them at appropriate positions in the WT EB1, finding that these substitutions enhanced protein’s LLPS capacity, as indicated by lower saturation concentration of the modified protein (Fig. 3*A*). Finally, we created a hybrid protein, EB3(NTD)-EB1(IDR)-EB1(CTD), in which the entire NTD of EB1 was replaced with that of EB3, and tested its ability to undergo LLPS. This hybrid protein exhibited greater capacity for LLPS compared to both full-length EB1 and the EB1 NTD variant (3HNTD) (Fig. 3*A*), consistent with the notion that histidine residues (Q29H, A57H, Q73H) in the NTD play an important role in promoting LLPS. However, somewhat lower c_sat_ of the EB3(NTD)-EB1(IDR)-EB1(CTD) hybrid protein compared to that of the 3HNTD EB1 variant also suggest that additional residues within the NTD likely contribute to LLPS, albeit to a lesser extent. Collectively, these data indicate that higher LLPS propensity of EB3 as compared to EB1 is due to collective contributions of all three domains, with His residues in the NTD playing a particularly important role.

Finally, given that EB3 droplets are less dynamic than those formed by EB1, we asked whether these differences in material properties could be reproduced using the hybrid protein EB1(3HNTD)-EB3(LCD-CTD). To probe this issue, we performed FRAP experiments, finding that the dynamicity of EB1(3HNTD)-EB3(LCD-CTD) droplets closely resembles that of EB3 droplets (Fig. 3*D*).

### EB3 condensates have high capacity to recruit tubulin and can nucleate microtubule formation

**Figure 4:**
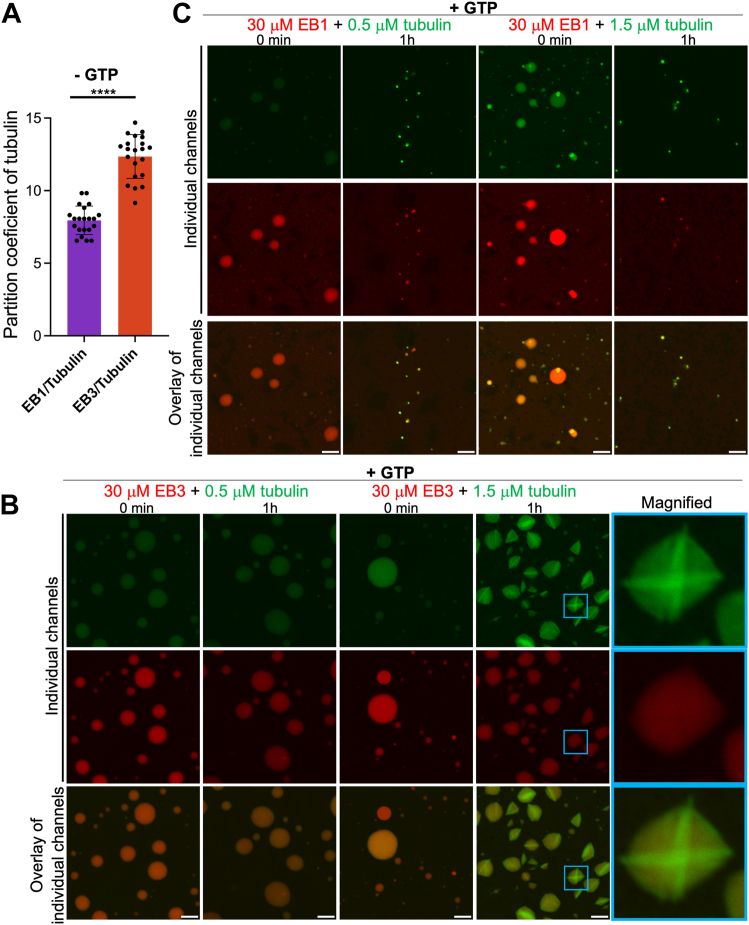
**EB3 droplets have higher capacity than EB3 droplets to recruit tubulin and nucleate microtubules.***A*, partition coefficients for tubulin (0.5 μM) into EB1 and EB3 droplets. The concentration of EB1 and EB3 in these experiments was 30 μM. Data represent averages for at least 21 droplets; *error bars* represent SD. Statistical significance was assessed using unpaired two-tailed Student’s *t* test. ∗∗∗∗*p* < 0.0001. No nucleotides were present in these experiments. *B* and *C*, representative fluorescence microscopy images of EB1 (panel *C*) and EB3 (panel *B*) droplets in the presence of tubulin and 1 mM GTP. Individual fluorescence microscopy channels and their overlays show images immediately after tubulin addition and after 1 h incubation in the presence of 1 mM GTP. Images in the last raw in panel *B* represent magnified images of droplets within blue squares in the second last raw. EB1 and EB3 were labeled with Alexa Fluor 594 (*red*), with a labeled-to-unlabeled protein ratio of 1:10. Tubulin was labeled with HiLyte 488 (*green*). Scale bar represents 5 μm.

It was previously reported that EB1 droplets can recruit tubulin into their interior (15). Here we assessed the relative capacity of EB1 and EB3 in this regard, finding the partition coefficient of tubulin between the dilute phase and EB3 droplets to be higher than that for EB1 droplets, indicating higher concentration of tubulin within EB3 condensates (Fig. 4*A*).

To assess potential functional consequences of this preferential tubulin recruitment into EB3 condensates, we repeated these experiments in the presence of GTP, a nucleotide needed for microtubule formation. At the concentration used in our experiments (0.5–1.5 μM), tubulin alone did not self-assemble into microtubules (Fig. S9*B*). However, when tubulin was added to preformed EB3 droplets, we observed tubulin polymerization within droplets’ interior (but not within the dilute phase) (Fig. 4*B*). In sharp contrast, no tubulin polymerization was observed when tubulin was added to preformed EB1 droplets, even at a concentration as high as 1.5 μM. Instead, in the presence of tubulin, EB1 droplets started to shrink, becoming smaller with time (Fig. 4*C*).

Overall, these results indicate major differences between EB1 and EB3 droplets with regard to their ability to recruit tubulin and show that tubulin within EB3 condensates (but not within EB1 condensates) can spontaneously polymerize into filaments. This is likely because tubulin becomes highly concentrated within EB3 droplets, reaching the concentration surpassing the critical threshold needed for nucleation reaction.

### Cocondensation of EB proteins with CLIP-170∗

+TIP bodies are multicomponent cellular condensates that, in addition to EBs, contain other proteins. One of the most important players here is CLIP-170 (16). As a step towards reconstitution and characterization of the entire EB1/CLIP-170/tubulin and EB1/CLIP-170/tubulin systems, first we characterized the LLPS behavior of the mixture of CLIP-170 with EB1 and EB3 in the absence of tubulin. Due to the difficulties in purification of larger quantities of full-length CLIP-170, these studies were performed using a critical N-terminal fragment of this protein, Δ351 to 1438 CLIP-170 (for simplicity referred to as CLIP-170∗) which contains the binding sites for both EBs and tubulin (4, 26). Even though the LLPS propensity of this fragment is much lower than that of the full-length protein (16), it still undergoes condensation *in vitro* at higher concentrations in the presence of crowding agents (Fig. S8).

**Figure 5:**
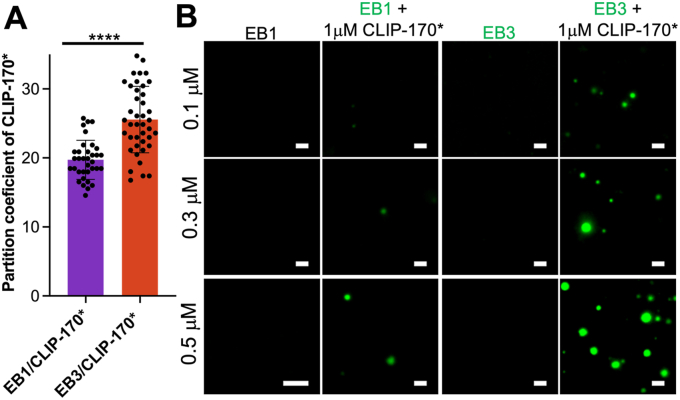
**EB1 and EB3 differentially cocondense with CLIP-170∗.***A*, partition coefficients for CLIP-170∗ (0.5 μM) recruitment into EB1 and EB3 droplets. The concentration of EB proteins was 30 μM. Data represent averages for at least 35 droplets. Error bars represent SD. Statistical significance was assessed using unpaired two-tailed Student’s *t* test. ∗∗∗∗*p* < 0.0001. *B*, representative fluorescence microscopy images of droplets formed by mixtures of EB proteins with CLIP-170∗ at sub-saturation concentrations of individual proteins. The concentration of EB1 and EB3 are indicated at the *left* side of each row. EB1 and EB3 were labeled with Alexa Fluor 488, and the ratio of labeled to unlabeled protein was 1:10 in each case. The scale bar represent 2 μm.

While both EB1 and EB3 have been shown to cocondense with the full-length CLIP-170 (16) or CLIP-170∗ (15), these studies were performed at different conditions and using different approaches, precluding comparison of relative capacities of EB1 and EB3 in this regard. Here we have explored this issue, finding only small differences between partition coefficients of CLIP-170∗ into preformed EB1 and EB3 droplets (20 and 25, respectively) (Fig. 5*A*). However, major differences between EB1 and EB3 were found with regard to their ability to cocondense with CLIP-170∗ when both proteins were below saturation concentration. Indeed, when CLIP-170∗ at 1 μM was mixed with as little as 0.3 μM EB3 (*i.e.*, well below saturation concentration of both proteins), there was robust formation of liquid droplets, indicating strong synergistic action of these two proteins (Fig. 5*B*). By contrast, much weaker, if any, synergy was observed for the mixture of CLIP-170∗ with EB1 (Fig. 5*B*).

### Tubulin recruitment into and microtubule formation within CLIP-170∗ droplets

**Figure 6:**
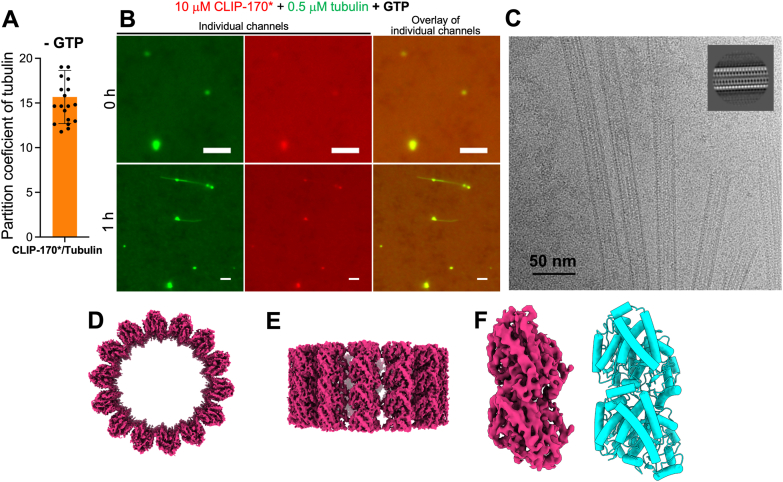
**CLIP170∗ droplets recruit tubulin and nucleate formation of microtubules.***A*, partition coefficient of tubulin (0.5 μM) into CLIP-170∗ droplets. The concentration of CLIP-170∗ was 20 μM. Data represents averages of 18 droplets; *error bars* represent SD. No nucleotides were present in these experiments. *B*, representative fluorescence microscopy images of CLIP-170 droplets in the presence of tubulin immediately (*top*) and 1 h after (*bottom*) addition of GTP (1 mM). CLIP-170∗ was labeled with Alexa Fluor 594 (*red*), with a labeled-to-unlabeled protein ratio of 1:10. Tubulin was labeled with HiLyte 488 (*green*). The scale bar represent correspond to 2 μm. *C*, a representative cryo-EM micrograph for tubulin filaments nucleated by CLIP-170∗ droplets in the presents of GTP (1 mM). Concentration of CLIP-170∗ and tubulin in this sample was 10 and 0.5 μM, respectively. The sample was incubated for 1 h, deposited on the grid and immediately frozen (see Experimental procedures for details). The micrograph shows filamentous structure resembling microtubules. two-dimensional class average (inset) further confirms the presence of a multi-protofilament structure. *D*, *top* view of the reconstructed 3D map of filaments reveals a 14-protofilament architecture. *E*, *side* view of the reconstructed 3D map of the filaments reveals that each protofilament is composed of stacked globular subunits. *F*, a stack of two subunits (*left*) within each protofilament resembles structural characteristics of a tubulin heterodimer in microtubules (*right*) (PDB 6e7c) (27), further supporting that these filaments are *bone fide* microtubules.

Next, we assessed the ability to CLIP-170∗ droplets to recruit and concentrate tubulin, finding that the latter protein is efficiently recruited to these droplets, with a partition coefficient of 17 (Fig. 6*A*). We also found that, when experiments were performed in the presence GTP, CLIP-170∗ condensates acted as nucleation sites for tubulin polymerization, with filaments often extending from the condensates (Fig. 6*B*). The ability of CLIP-170∗ condensates to nucleate tubulin polimerization was observed at tubulin concentration as low as 0.5 μM (Fig. 6*B*).

It should be noted that the tubulin polymerization capacity of CLIP-170∗ condensates is significantly higher than that of condensates formed by EB proteins. Indeed, no such nucleation was observed in our study for EB1 droplets. EB3 droplets, on the other hand, could nucleate tubulin polymerization, but only in the presence of a three-fold higher concentration of tubulin than it was the case for CLIP-170∗ droplets (Fig. 3*B*). Furthermore, filaments within CLIP-170∗ droplets appeared morphologically different from those nucleated by EB3 droplets (Figs. 3*B* and 5*B*). While the latter were localized exclusively within the droplet phase, filaments nucleated by CLIP-170 condensates were generally much longer, with their tips decorated with the condensates.

To gain insight into the nature of these filaments, we performed cryo-electron microscopy (cryo-EM). Analysis of cryo-EM micrographs revealed filamentous structures closely resembling microtubules and two-dimensional class averages further confirmed the presence of a multi-protofilament organization (Fig. 6*C*). A top view of the reconstructed 3D map shown in Figure 6*D* reveals a 14-protofilament architecture, while the side view (Fig. 6*E*) reveals that each protofilament is composed of stacked globular subunits. Even though at this stage the resolution of our reconstitution is still relatively low (6.4 Å), the structure of these subunits (Fig. 6*F*) closely resembles that of tubulin heterodimers found in microtubules (PDB 6E7C) (27), clearly demonstrating that the observed filaments are indeed *bona fide* microtubules.

### Cocondensates of EB3 and CLIP-170 nucleate microtubules at an order of magnitude lower concentration of tubulin than cocondensates of EB1 and CLIP-170

**Figure 7:**
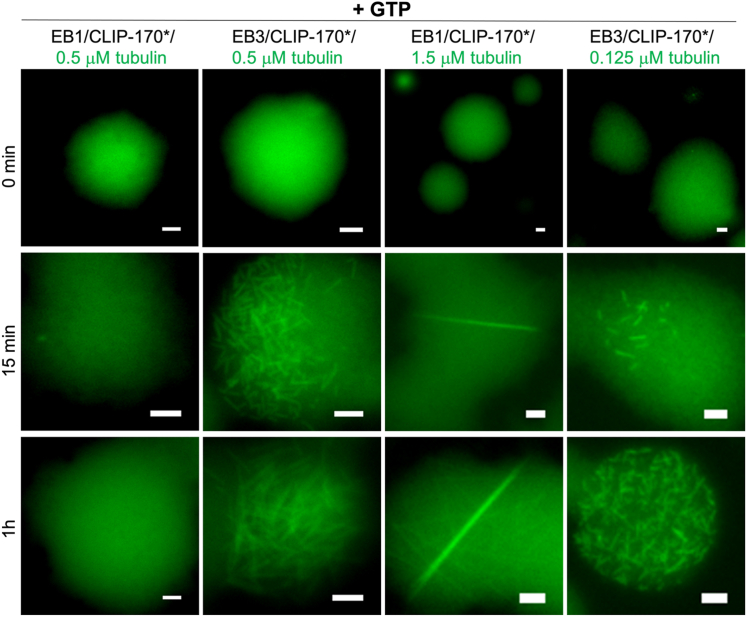
**Cocondensation of EB1/CLIP-170∗ and EB3/CLIP-170∗ with tubulin.** Representative fluorescence microscopy images of EB1/CLIP-170∗ and EB3/CLIP-170∗ droplets in the presence of tubulin immediately *(top row*), 15 min (*middle row*), and 1 hour (*bottom row*) after the addition of GTP (1 mM). The concentration of EB1 and EB3 was 20 μM and that of CLIP-170∗ was 10 μM. Tubulin was labeled with HiLyte 488 (*green*). The scale bar represent, 2 μm.

Given that EB proteins and CLIP-170∗ can co-condense into miscible droplets (Fig. 5), finally we explored whether there is a synergistic action of these two key components of the +TIP network in nucleation of tubulin polymerization. To this end, we mixed EB1 or EB3 with CLIP-170∗ in the presence of 0.5 μM tubulin. In both cases, all components were miscible within the droplet phase (Fig. S9*A*). Upon addition of GTP, EB3/CLIP-170∗ droplets in the presence of 0.5 μM tubulin were found to nucleate formation of filaments (Fig. 7). Remarkably, this nucleation activity of EB3/CLIP-170∗ droplets was observed even at tubulin concentration as low as 0.125 μM (Fig. 7). The behavior of EB1/CLIP-170∗ droplets was quite different, with no nucleation of tubulin polymerization observed in the presence of 0.5 μM tubulin, even though thick filaments could be seen upon increasing tubulin concentration to 1.5 μM (Fig. 7). Taken together, these data indicate that cocondensates of CLIP-170∗ with EB3 nucleate tubulin polymerization at an order of magnitude lower tubulin concentration than the cocondensates of CLIP-170∗ and EB1.

**Figure 8:**
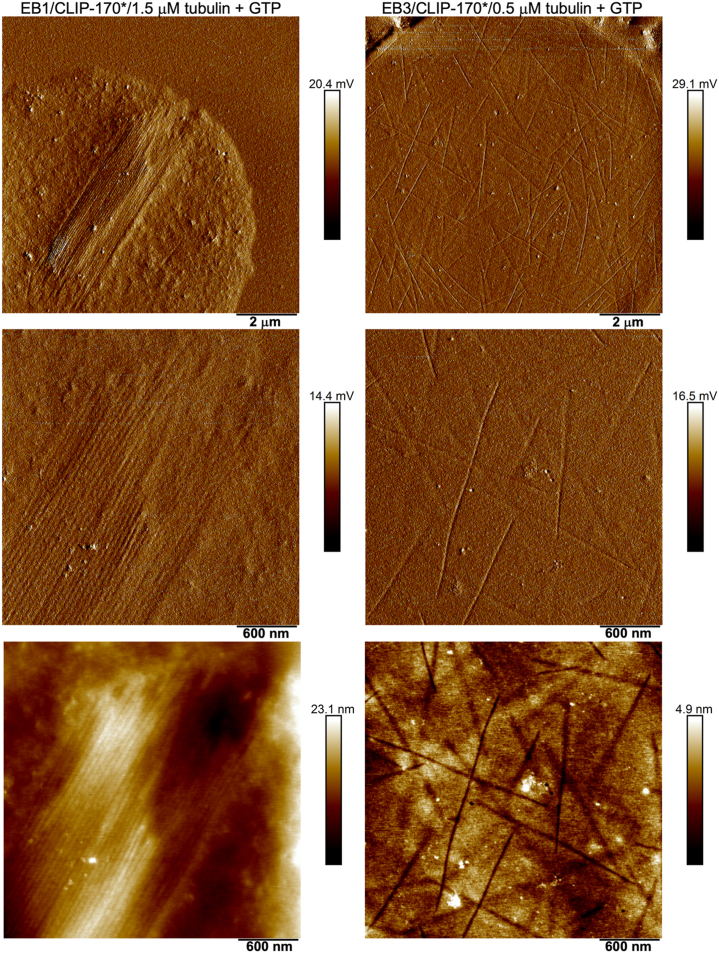
**Fibrillar nature of tubulin polymers formed within EB1/CLIP-170∗ and EB3/CLIP-170∗droplets.** Representative atomic force microscopy peak force error images *(top* and *middle rows*) and height images (*bottom row*) show filaments formed by tubulin within EB1/CLIP-170∗ and EB3/CLIP-170∗droplets after 1 h incubation in the presence of GTP (1 mM). *Middle* and *bottom row* images represent magnified views of the *top* row images. The concentration of EB1 and EB3 was 20 μM and that of CLIP-170∗ was 10 μM. Tubulin was labeled with HiLyte 488 (*green*).

In contrast to tubulin filaments nucleated within droplets formed by CLIP-170∗ alone (that extended beyond the condensates), efforts to use cryo-EM for structural analysis of tubulin polymers formed within EB1/CLIP-170∗ or EB3/CLIP-170∗ droplets have not yet been successful. Therefore, to gain some insight into the nature of the latter, we resorted to a lower-resolution method of atomic force microscopy (AFM). Analysis of AFM images of samples dried on mica surface (Fig. 8) confirmed light microscopy-based notion that both types of condensates are capable of nucleating filament formation, albeit at different tubulin concentration. These images also revealed that filaments formed within EB3/CLIP-170∗ condensates appear as individual, randomly distributed fibrils (right panels in Fig. 8) By contrast, filaments nucleated within EB1/CLIP-170∗ condensates appear to self-associate laterally, forming thick bundles (left panels in Fig. 8). Unfortunately, a strong background from other proteins in dried droplets (EB1/EB3 and CLIP-170∗) precluded reliable measurements of filaments’ height in AFM images, leaving open the question whether these filaments also represent *bona fide* microtubules.

**Figure 9:**
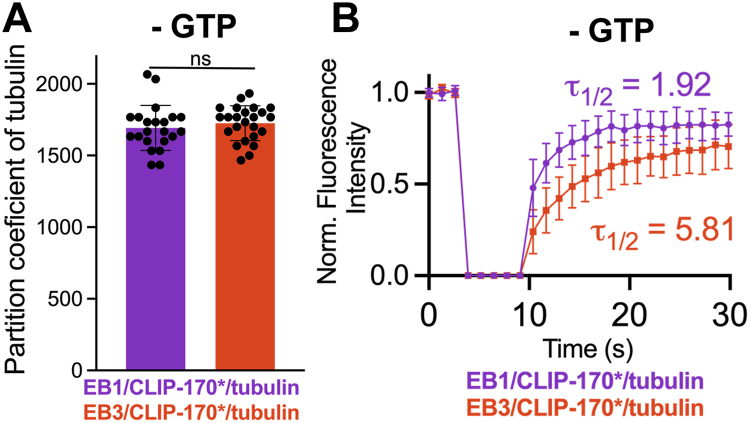
**Distinct material properties of EB1/CLIP-170∗/tubulin and EB3/CLIP-170∗/tubulin condensates.***A*, partition coefficient of tubulin into EB1/CLIP-170∗ and EB3/CLIP-170∗ droplets in the absence of any nucleotides. The concentration of EB1 and EB3 was 20 μM and that of CLIP-170∗ was 10 μM. Data represent averages for at least 22 droplets; *error bars* represent SD. Statistical significance was assessed using unpaired two-tailed Student’s *t* test; ns - nonsignificant (*p* > 0.05). *B*, representative FRAP traces for HiLyte 488-labeld tubulin in EB1/CLIP-170∗/tubulin and EB3/CLIP-170∗/tubulin droplets in the absence of any nucleotides. Each trace represents the average of measurements for 7 droplets; error bars represent SD. FRAP, fluorescence recovery after photobleaching.

Since EB3 droplets alone recruit tubulin more efficiently than EB1 droplets (Fig. 4*A*), we hypothesized that higher capacity of EB3/CLIP-170∗ condensates than that of the EB1/CLIP-170∗ counterparts to nucleate tubulin polymerization could be due to higher tubulin recruitment ability of the former. Surprisingly, however, we found that the partition coefficient of tubulin to the two droplet types was very similar, in both cases remarkably high (Fig. 9*A*). Thus, the observed major differences in microtubule nucleation efficiency of EB3/CLIP-170∗ and EB1/CLIP-170∗ droplets appear to be due factors other than differences in tubulin concentration within the condensed phase. In search of such potential factors and guided by our initial observation that EB3 droplets are less dynamic than the EB1 counterparts (Fig. 3*A*), we performed FRAP measurements with HiLyte 488-labeled tubulin to examine material properties of EB1/CLIP-170∗/tubulin and EB3/CLIP-170∗/tubulin condensates. Our results revealed that, even though the mobile fraction of tubulin in both condensate types was similar, the half-time of fluorescence recovery after photobleaching within EB3/CLIP-170∗ droplets was approximately threefold longer than that within EB1/CLIP-170∗ droplets (Fig. 9*B*), indicating a more viscous internal environment in EB3-containing condensates. Based on these data, we propose that the differences between EB1/CLIP-170∗/tubulin and EB3/CLIP-170∗/tubulin condensates with regard to their capacity to nucleate tubulin polymerization may be due to distinct material properties of these condensates. As elaborated in the Discussion section below, the general idea that solvent viscosity could control the nucleation rate of tubulin polymerization is consistent with previous observations in the absence of phase separation (28, 29, 30).

## Discussion

Mammalian cells express three end binding proteins—EB1, EB2, and EB3—that, despite being encoded by separate genes (31), are structurally similar, with 57 to 67% amino acid identity (7). Most studies on the function of EBs in mammalian cells and in the test tube have primarily focused on EB1 or EB3, while the role of EB2 remains largely unexplored. These studies have revealed, among others, that both EB1 and EB3 interact with many + TIP proteins such as CLIP-170, CLIP-115, and MCAK (32, 33). Furthermore, it was shown that these two end binding proteins control CLIP localization at the growing ends of microtubules (32) and promote persistent microtubule growth by suppressing catastrophes (34).

These similarities notwithstanding, there are a number of important differences between EB1 and EB3 with regard to their interaction with specific protein partners. For example, EB3, but not EB1, interacts with the F-actin-binding protein drebrin, and this interaction is crucial for the formation of the growth cone and neuritogenesis (35). Furthermore, EB3, but not EB1, interacts with the E3 ubiquitin ligase SIAH-1 (36). It was also shown that, during mitosis, two kinases, Aurora-A and Aurora-B, phosphorylate selectively EB3. This, in turn, prevents EB3 degradation by SIAH-1, thereby increasing its concentration and facilitating cell cycle progression (36).

Recently, EBs have been shown to undergo LLPS both in the test tube and in cells, and it has been suggested that condensation of these proteins may play an important role in the biogenesis and functional properties of microtubules (15, 16, 21, 22). Given the apparent differences between the members of the EB family with regard to some of their functional properties, here we focused on detailed comparison of the phase separation behaviour of EB1 and EB3. Our experiments in the test tube and in cells revealed that EB3 has markedly higher LLPS propensity than EB1. We also found that protein concentration within EB3 droplets is substantially higher than that within EB1 droplets, and that condensates formed by EB3 have much higher capacity to recruit tubulin.

Previous studies with EB1 suggested that LLPS of the latter protein is driven by electrostatic interactions between different domains, with Arg and Lys residues in the IDR playing a key role (15). Our present findings regarding major differences in LLPS capacity between EB1 and EB3 are quite surprising in light of these previous studies, as both proteins have similar content of positively charged residues within their IDRs. Upon further investigation, we concluded that these differences are due to collective contributions of all protein domains, with three His residues present in the NTD domain of EB3 (but absent in EB1) playing a particularly important role.

The present findings regarding differences in the LLPS behavior of EB1 and EB3 may be of direct relevance to previous observations regarding the properties of microtubules. In particular, it was reported that EB3 comets at microtubule ends are much brighter as compared to EB1 comets (37). Based on our data, we propose that this could be, at least in part, due to higher LLPS capacity of EB3 and the resulting higher concentration of the latter protein within the condensed phase. The same previous study also found that, compared to EB1, EB3 is more potent at increasing microtubule growth rate (37). Our findings regarding greater tubulin recruitment capacity of EB3 droplets suggest that this difference could be related to higher tubulin concentration within EB3 condensates than EB1 condensates, as this would lead to faster microtubule growth. It should be noted in this context that, unlike highly specific binding of EB proteins to microtubules *via* the interface between four tubulin subunits (2, 38), the recruitment oof tubulin dimers into EB droplets is likely driven by weak, relatively nonspecific interactions between these two proteins.

Another recent study proposed that material properties of +TIP bodies that decorate microtubules may play a role in their functional specialization (19). Specifically, these properties—including wetting behavior, client partitioning, surface tension, and viscosity—have been suggested to affect the growth or shrinkage of microtubules, induce symmetry breaking, couple forces between microtubules and other structures, and regulate microtubule-based transport to specific destinations (19). Our present findings regarding distinct material properties of EB1 and EB3 condensates are particularly relevant in this context, suggesting that + TIP bodies-dependent microtubule specialization could depend on the type of EB protein associated with these bodies. Further studies are required to explore this possibility.

Finally, we found that droplets formed by CLIP-170∗ alone or those formed by the mixture of CLIP-170∗ and EB proteins can efficiently nucleate polymerization of tubulin, with at least some of these polymers having structural characteristics of *bona fide* microtubules. Importantly, significant differences with regard to this polymerization nucleation capacity were observed between EB1/CLIP-170∗ and EB3/CLIP-170∗ droplets. In particular, we found that an order of magnitude lower total concentration of tubulin is required to nucleate its polymerization in the presence of EB3/CLIP-170∗ droplets than it is the case in the presence of EB1/CLIP-170∗ droplets. This is not due to differences in tubulin concentration within the condensed phase, as EB1/CLIP-170∗ and EB3/CLIP-170∗ droplets concentrate tubulin to a similar extent. Thus, we propose that differences in droplets’ material properties may play a major role as a modulator of tubulin polymerization. The above hypothesis is consistent not only with our findings regarding viscosity differences within EB1/CLIP-170∗ and EB3/CLIP-170∗ droplets, but also with previous observations in the absence of LLPS. The latter indicated that solvent viscosity has a major effect on microtubule nucleation, with increased viscosity facilitating the nucleation reaction while, at the same time, reducing microtubule elongation rate (29, 30). These findings are at present limited to experiments *in vitro*, and it remains to be determined whether similar mechanisms are operational in a cellular context. Nevertheless, it is worth noting that the capacity to concentrate tubulin and nucleate its polymerization into microtubules has been proposed for several other LLPS-prone MAPs, including tau (39), the chromosomal passenger complex (40), TPX2 (41), MAP65-1 (42), and NuMA (43). These examples collectively suggest that concentrating tubulin within liquid-like condensates of cytoskeletal regulatory proteins may represent a general mechanism for the formation of local microtubule nucleation centers. The types of microtubule arrays that arise from these condensates (*e.g.*, bundles, asters, parallel arrays, or individual filaments) are likely to depend on the type of MAPs that form the droplets (39).

## Experimental procedures

### Expression, purification, and labeling of recombinant proteins

Genes encoding human EB1 (UniProt ID: Q15691), human EB3 (UniProt ID: Q9UPY8), their hybrid variants, and human Δ351 to 1438 CLIP-170 (denoted Clip-170∗) (UniProt ID: P30622) were synthesized by Genscript and subcloned into the pET-15b. All variants contained N-terminal His-tag and thrombin cleavage site sequences. Proteins were expressed in Rosetta *Escherichia coli*. Bacteria were grown at 37 °C and harvested 1.5 h after induction with 0.75 mM isopropyl 1-thio-β-D-galactopyranoside. EB proteins and their hybrid variants were purified on an immobilized nickel-affinity column (Ni-NTA Fast Flow, Qiagen). The post-FPLC protein was diluted by a factor of 1.4 into a 10 mM Hepes (pH 7.4) solution. Immediately following this step, biotinylated thrombin was added (Novagen; 0.6 units/mg protein), and the solution was incubated overnight at ambient temperature. Thrombin was then captured using streptavidin-agarose beads. Cleaved protein was further diluted by a factor of 20 into a 10 mM Hepes (pH 7.4) solution. This was followed by further purification on an anion-exchange column (Mono-Q, GE Healthcare) using a linear gradient of NaCl. Purified proteins were dialyzed against 10 mM Hepes (pH 7.4) containing 300 mM KCl. Δ351 to 1438 CLIP-170 was purified using immobilized nickel-affinity chromatography and cleaved with biotinylated thrombin as described above. Following thrombin capture, the cleaved protein was buffer-exchanged into 10 mM Hepes (pH 7.4) containing 500 mM NaCl using Zeba spin desalting columns (Thermo Fisher Scientific). The cleaved His-tag was subsequently removed by incubation with Ni-NTA Fast Flow resin. The protein was then dialyzed against 10 mM Hepes (pH 7.4) containing 300 mM KCl and 1 mM DTT. All cleaved proteins in the present study contained three additional N-terminal residues (GSH). The purity of all proteins was assessed by SDS-PAGE (Fig. S10). Protein concentration was determined by absorbance at 280 nm using extinction coefficients calculated based on amino acid composition. Proteins were labeled with Alexa Fluor 488 or 594 C5 Maleimide, or 647 C2 Maleimide (Invitrogen), by adding 10 μl of the dye in DMSO (10 mg/ml) to 100 μl of the protein (6–9 mg/ml) in 10 mM Hepes (pH 7.4) containing 300 mM KCl, and incubating the mixture at room temperature for 2 h with stirring. Excess dye was removed using Zeba spin desalting columns (Thermo Fisher Scientific). Labeling efficiency was estimated based on the relative concentrations of the protein and the dye according to the manufacturer’s instructions.

### Monitoring of LLPS

Phase separation experiments involving EB1, EB3, their hybrid variants, and CLIP-170∗ were conducted under physiologically relevant conditions in 10 mM Hepes buffer (pH 7.4) containing 100 mM KCl, 1 mM DTT, and 8% PEG-10 (unless indicated otherwise). Experiments in the presence of tubulin were performed in a BRB80 buffer (80 mM PIPES (pH 6.8), 1 mM MgCl_2_, 1 mM EGTA) that is typically used in studies on microtubule formation. This buffer was supplemented with 1 mM DTT, 34 mM KCl, and 8% Dextran-70. GTP (1 mM) was added in some experiments. LLPS was monitored by turbidity (absorbance at 400 nm corrected for the absorbance of buffer blank) at 25 °C using the M1000 Tecan plate reader. To determine saturation concentration (C_sat_) of individual proteins, these proteins were added at increasing concentrations to a solution containing 10 mM Hepes (pH 7.4), 100 mM KCl, 1 mM DTT, and 8% PEG-10, and OD at 400 nm was measured for each sample. These OD values (corrected for a buffer blank) were then plotted as a function of protein concentration and these data were fitted with two linear functions: one in the low (essentially flat) OD region (corresponding to the absence of LLPS) and one in the region showing a gradual increase in OD (corresponding to LLPS). The concentration at the intersection of these two lines corresponds to C_sat_. To obtain phase diagrams, samples were prepared as described above, except that a range of PEG concentrations (2–10%) was used. Protein concentration *versus* PEG concentration phase diagrams were constructed based on turbidity measurements, with boundaries of phase separation defined as OD at 400 nm of ≥ 0.02 (what corresponds to threshold OD that could be reproducibly determined in these experiments). LLPS in the boundary regions was verified by fluorescence microscopy. All these measurements were performed within 5 − 10 min of sample preparation.

### Fluorescence microscopy imaging of droplets formed *in vitro*

Droplets were visualized by fluorescence microscopy using Alexa Fluor 488-, Alexa Fluor 594-, or Alexa Fluor 647-labeled proteins that were mixed with unlabeled proteins at a molar ratio of 1:10. In experiments involving tubulin, HiLyte 488-labeled tubulin (Cytoskeleton) was used without dilution with unlabeled protein. Samples (20 μl) were placed on the glass bottom of a 35-mm dish that was covered with a microscope cover glass. Microscopy experiments were performed at room temperature on a Keyence BZ-X710 microscope with a × 100/1.45 numerical aperture oil-immersion lens. Partition coefficients were calculated as the ratio of fluorescence intensity of protein within the droplet phase to that in the dilute phase. In experiments involving mixtures of two or more proteins, only the protein for which the partition coefficient was being determined was labeled. In these experiments, droplets were allowed to sediment at the bottom of a 35-mm dish for 15 min prior to imaging on Leica HyVolution SP8 confocal microscope with × 63/1.4 numerical aperture oil-immersion objective. To obtain the partition coefficient, the fluorescence intensity within the region of interest at the center of the droplet was divided by the fluorescence intensity of an identically sized region of interest within the background far away from the droplet. For experiments in the presence of GTP (1 mM), sample was incubated in the test tube for the indicated periods of time and then placed at the bottom of a 35-mm dish and visualized by fluorescence microscopy.

### FRAP experiments

Samples for FRAP were prepared as described above for fluorescence microscopy imaging experiments. Droplets were allowed to sediment at the bottom of a 35-mm dish for 15 min prior to imaging. FRAP measurements were performed using a Leica HyVolution SP8 confocal microscope with 2.4-mW laser intensity for bleaching, × 63/1.4 numerical aperture oil-immersion objective, and photomultiplier tube detector. For the experiments with EB1, EB3, and their hybrid variants, the regions of interest (0.4-μm diameter) were bleached within ∼2.5 μm droplets. The measurements involved three pre-bleaching frames, six flashes of bleaching (65% of laser power), and 70 post-bleaching frames (1.3 s/frame). For the experiments with EB1/CLIP-170∗/tubulin and EB3/CLIP-170∗/tubulin, the regions of interest (0.33-μm diameter) were bleached within ∼2.5 μm droplets. The measurements involved three pre-bleaching frames, five flashes of bleaching (50% of laser power), and 16 post-bleaching frames (1.3 s/frame). The latter FRAP measurements parameters were used to avoid photofading of labeled tubulin. Individual FRAP traces were analyzed by EasyFRAP (44). Raw data were normalized using double-scale normalization. The mean normalized curve with the standard deviation was plotted. The mean normalized curve was fitted using a double exponential equation to calculate the values of mobile fraction and half-times of fluorescence recovery.

### Plasmids for expression in HeLa cells

Cry2WT-mCherry plasmid was produced by reversing the E490 G mutation of the Cry2olig-mCherry (Addgene, 60,032) backbone. Plasmids for Cry2WT-mCherry-EB1 and Cry2WT-mCherry-EB3 expression were prepared by inserting DNA fragments encoding EB1 or EB3 from pET15b-EB1 or pET15b-EB3, respectively, into the Cry2WT-mCherry plasmid. Subcloning was performed by Genscript.

### Cell culture and transfection

HeLa cells were obtained as a gift from Dr E. Jankowski and maintained in Dulbecco’s Modified Eagle Medium with 10% heat-inactivated fetal bovine serum and 1% Penicillin-Streptomycin at 37 °C in 5% CO_2_. Monolayers were passaged upon reaching 90 to 95% confluency using 0.05% trypsin protease. Cells were grown in a 35 mm glass-bottom dish coated with Poly-D-Lysine (Gibco) and allowed to reach ∼50% confluency before transient transfection. X-tremeGENE 9 (Roche) and Opti-MEM were used for transient transfection according to the manufacturer’s instructions with plasmids encoding Cry2WT-mCherry, Cry2WT-mCherry-EB1, and Cry2WT-mCherry-EB3. Cells were incubated for additional 24 h prior to imaging or lysis.

### Live cell imaging of optoDroplets and analysis of fluorescence microscopy images

Before imaging, cell culture medium was replaced with Dulbecco’s Modified Eagle Medium without phenol red. Live cell imaging was performed using an Olympus IX-81 Fluoview FV3000 confocal laser scanning system with a 60× 1.40 numerical aperture oil objective. Cells were imaged using two laser wavelengths: 488 nm for Cry2 activation (10% of 488-nm light power per 30 s) and 562 nm for mCherry visualization.

Microscopy image quantification was performed using Fiji software (https://imagej.net/software/fiji/downloads) (45). Since transient transfection of cells results in varying levels of transgene expression, the first step in our image analysis was calculation of corrected total cell fluorescence (CTCF) of mCherry labeled proteins, which was used as a measure of the level of protein expression (46, 47). mCherry fluorescence intensity in pre–blue light exposure images was calculated using the formula:

CTCF = Integrated Density – (Area of selected cell × Mean fluorescence of background)

Cells with CTCF in the range of 130,000 to 380,000 were selected for further analysis. To determine the number of droplets formed in these cells upon blue light stimulation, a single-plane image acquired before blue light exposure was subtracted from the corresponding single-plane image acquired after blue light exposure using the Image Calculator function in Fiji. The resulting difference image was inverted and adjusted using auto brightness/contrast followed by a mean filter (1-pixel radius). Image segmentation was then performed using the Max Entropy automatic threshold. The resulting binary images were analyses using the Analyze Particles function, where particles larger than 0.1 μm^2^ were counted as droplets. The number of droplets was then plotted as a function of CTCF.

### Immunoblotting

Cells were lysed in RIPA lysis and extraction buffer (Pierce) supplemented with a protease inhibitor cocktail (Pierce) and centrifuged at 13,000*g* for 10 min at 4 °C to collect the supernatant. Samples were then boiled in Laemmli sample buffer for 5 min. Proteins were separated by PAGE using NuPAGE 4 to 12% Bis-Tris gels (Invitrogen) and blotted onto Nitrocellulose membranes. Following blocking with 5% nonfat dry milk in TBS for 1 h, membranes were incubated for 3 h at room temperature with horseradish peroxidase-conjugated recombinant mCherry rat monoclonal antibody (16D7) (Invitrogen # 740003THRP20UG, 1:1000) in TBS blocking buffer. The specificity of this antibody was validated by the manufacturer. Membrane was imaged with SuperSignal West Pico Plus Chemiluminescence Substrate (Thermo Fisher Scientific) using the Azure 600 imaging system.

### Atomic force microscopy imaging

Samples were deposited onto freshly cleaved mica substrates, left at room temperature for 3 min, then washed three times with Milli-Q water and dried under nitrogen (N_2_). Images were acquired in a Scan Asyst mode using a silicon probe (spring constant: 40 N/m) on a Bruker MultiMode atomic force microscope equipped with a NanoScope V controller. Image processing was carried out using the NanoScope Analysis software.

### Cryo-EM experiments and data processing

*Data collection.* Single-layer chemical vapor deposition graphene (Graphenea, Spain) was transferred to three hundred mesh Quantifoil R 3.5/1 Holey Carbon grids (Quantifoil (48), and these grids were treated by UV Ozone cleaner (Ossila) for 7 min. Three microliters of a sample of tubulin filaments nucleated by CLIP-170∗ droplets was applied onto freshly prepared graphene grids, blotted for 5 seconds and plunge-frozen in liquid ethane using a Vitrobot Mark IV (Thermo Fisher Scientific). Movies were collected on a Titan Krios G3i microscope (Thermo Fisher Scientific) equipped with a BioQuantum K3 camera (Gatan=) with 0.414 Å/pixel in a super-resolution mode. A total of 40 movies were automatically collected using SerialEM (49).

*Data processing.* Movies were corrected for drifting and binned by a factor of 2 using the MotionCor2 algorithm implemented in RELION (50). Contrast transfer functions were estimated by CTFFIND v.4.1.3 (51). All further processing was carried out using RELION (52). Individual filaments were manually picked and overlapping segments were extracted using an interbox distance of 10 Å. Suboptimal segments were removed after two-dimensional classifications. In 3D classification, 3-start helical symmetries of microtubules with different numbers of protofilaments (27) were converted to 1-start helical symmetry and implemented. The helical symmetry of 14-protofilament microtubules (rise=2.92 Å, twist = −128.57°) significantly improved the overall resolution and, therefore, was used and further refined. The overall resolution of the final map was calculated to be 6.4 Å from Fourier shell correlations at 0.143 between two independently refined half-maps.

## Data availability

All data are contained within this article and the supporting information. CryoEM map of microtubules generated from tubulin partitioned into droplets of CLIP-170∗ has been deposited to the Electron Microscopy Data Bank (EMDB) with the accession code: EMD-72661.

## Supporting information

This article contains supporting information (53, 54).
