# Supplementary material for: Distinct liquid-liquid phase separation properties of end-binding proteins EB1 and EB3
Source: J Biol Chem. 2025 Oct 22;301(12):110849. doi: 10.1016/j.jbc.2025.110849 (PMC12661437; doi:10.1016/j.jbc.2025.110849)
Supplement: Supporting information [file mmc1.pdf]

Supporting information for

Distinct liquid-liquid phase separation properties of end-binding proteins EB1 and EB3

Solomiia Boyko, Qiuye Li, Krystyna Surewicz, and Witold K. Surewicz*

Department of Physiology and Biophysics, Case Western Reserve University, Cleveland, Ohio 44106

*Corresponding author. Email: wks3@case.edu

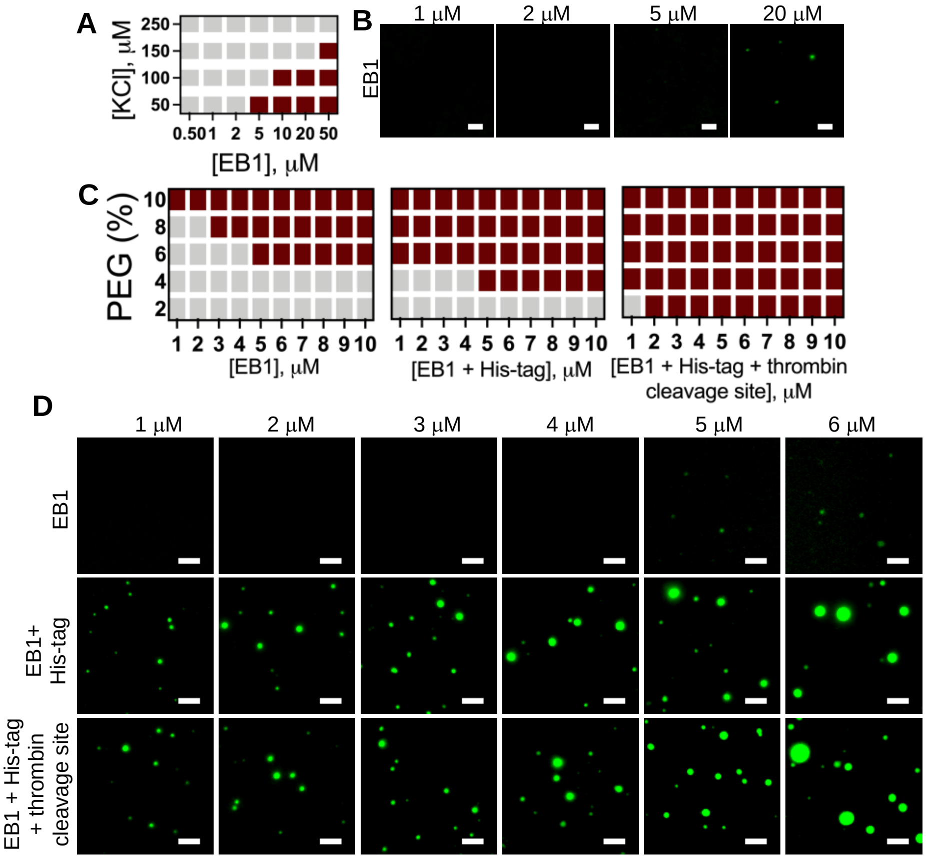

Figure S1. His-tag increases the propensity of EB1 to undergo LLPS. *A,* protein concentration versus salt concentration phase diagram for tag-free EB1 in BRB80 buffer (80 mM PIPES (pH 6.8), 1 mM $MgCl_2$, and 1 mM EGTA) without any crowding agent. Gray and brown boxes indicate the absence and presence of phase separation, respectively. *B*, Representative fluorescence microscopy images of tag-free EB1 in crowders-free BRB80 buffer containing 100 mM KCl. Protein concentrations are marked at the top of individual images. *C,* protein concentration versus PEG concentration phase diagrams for EB1 without and with different tags in 10 mM HEPES (pH 7.4) buffer containing 100 mM KCl and 1 mM DTT. Gray and brown boxes indicate the absence and presence of phase separation, respectively. *D,* representative fluorescence microscopy images of EB1 without and with different tags in the same buffer as above but containing 6% PEG. Protein concentrations are marked at the top of each column. The proteins were labeled with Alexa Fluor 488 (green), and the ratio of labeled to unlabeled protein was 1:10 in each case. Scale bars correspond to 2 μm.

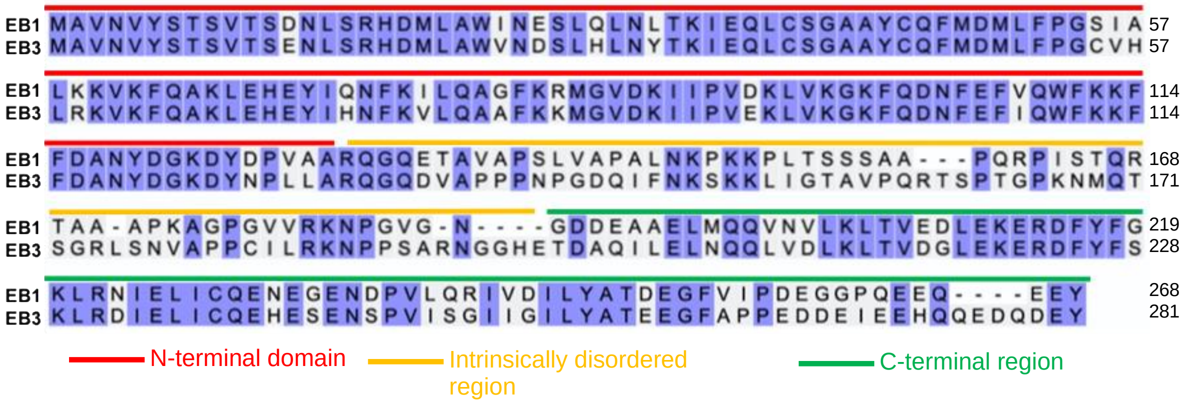

Figure S2. Sequence alignment of EB1 and EB3. Identical residues are highlighted in purple. Different protein domains are marked with color-coded lines.

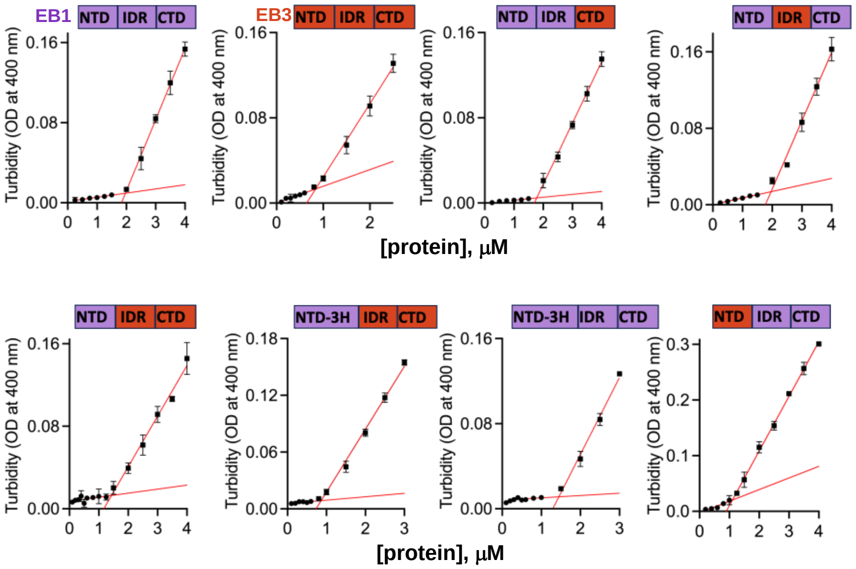

Figure S3. Determination of saturation concentrations (c_{sat}) for EB1, EB3 and the hybrid variants. Turbidity (OD at 400 nm) was monitored as a function of protein concentration. The concentrations at which the two straight lines drawn through experimental data points intersect correspond to c_{sat}. Error bars represent SD (n=3-7).

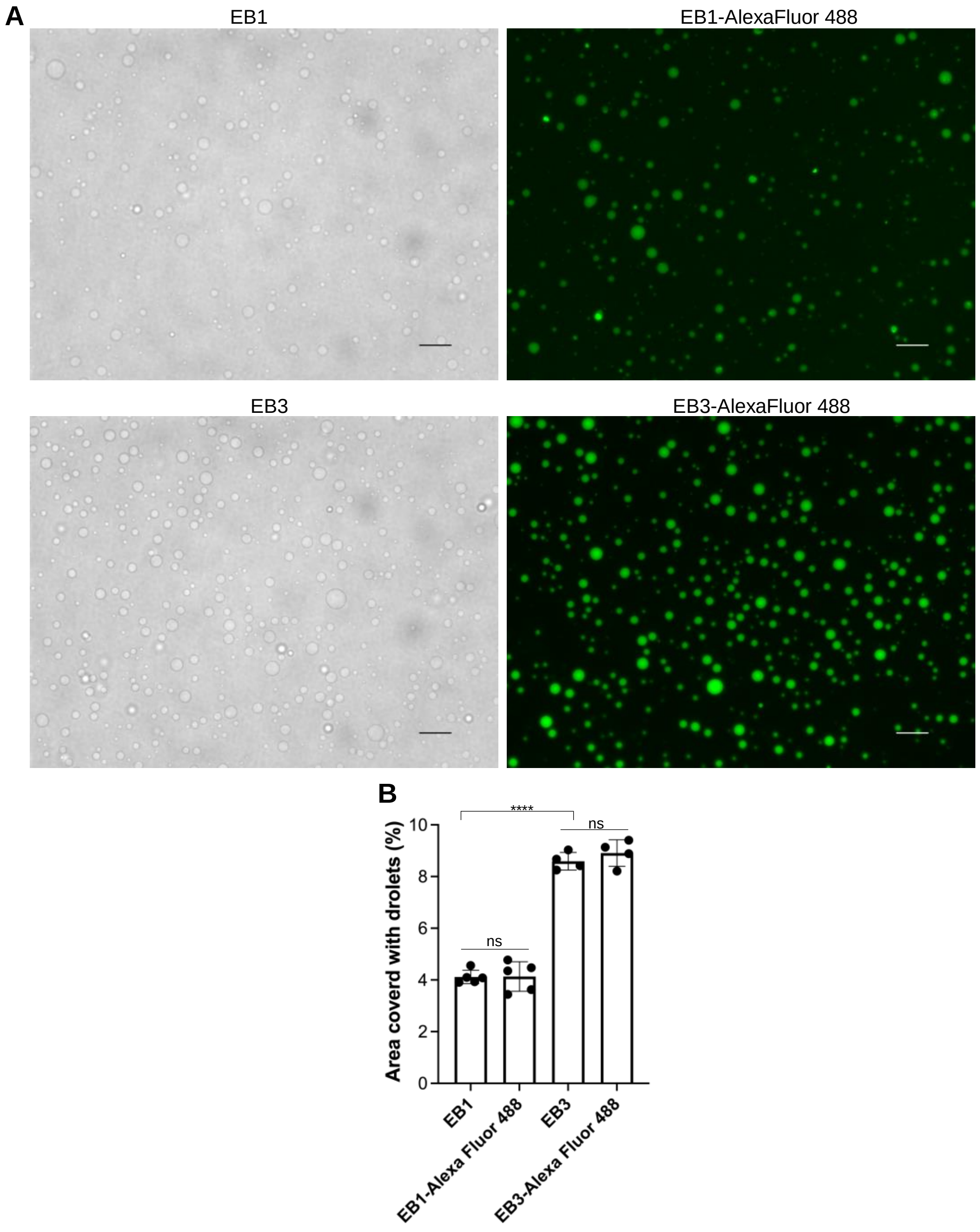

Figure S4. *A*, representative bright field and fluorescence microscopy images of droplets formed by EB1 or EB3 (10 µM each). For bright field imaging, only unlabeled proteins were used. For fluorescence microscopy, proteins were labeled with Alexa Fluor 488 (green) at a 1:10 ratio of labeled to unlabeled protein in each case. Scale bars: 10 µm. *B,* surface area covered by droplets formed by either unlabeled or Alexa Fluor 488-labeled EB1 or EB3 (10 µM each). Data represent averages of at least four fields, 15948 μm^2 each; error bars represent SD. Statistical significance was assessed using unpaired two-tailed Student's t-test. **** $P < 0.0001$, ns - non-significant.

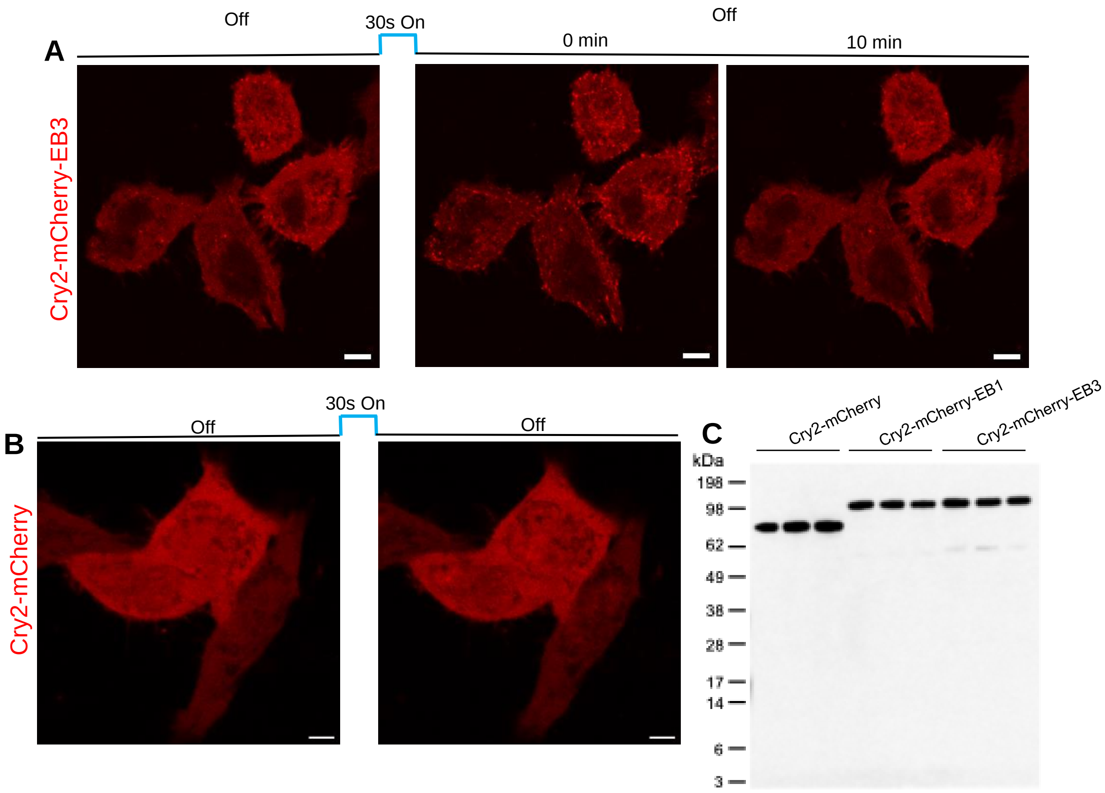

Figure S5. *A*, representative fluorescence images of blue light-activated assembly of Cry2-mCherry-EB3 in HeLa cells show that EB3 forms dynamic droplets in HeLa cells, which disassemble 10 minutes after light exposure. Scale bar corresponds to 5 µm. *B*, representative fluorescence images of Cry2-mCherry control in HeLa cells. As expected, no droplets are formed upon exposure to blue light. Scale bar corresponds to 5 µm. *C*, western blot analysis of total cell lysates of HeLa cell expressing Cry2-mCherry (left three lanes), Cry2-mCherry-EB1 (middle three lanes), or Cry2-mCherry-EB3 (right three lanes). Blots were probed with an antibody against mCherry.

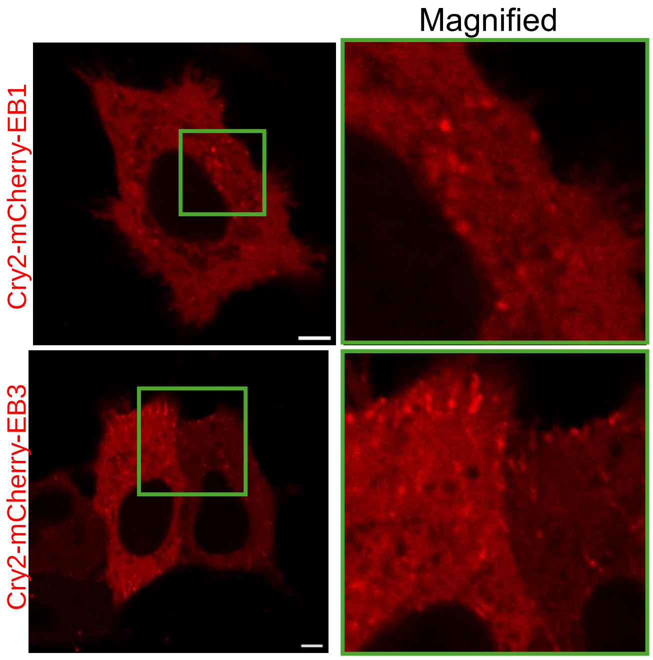

Figure S6. Representative fluorescence images showing granules formed by Cry2-mCherry-EB1 (top) and Cry2-mCherry-EB3 (bottom) in HeLa cells in the absence of blue light stimulation. These granules were observed only in a small population of cells with very high expression level of these proteins (corrected total cell fluorescence intensity of 920,000 and 1,100,000 A.U. for the cell expressing Cry2-mCherry-EB1 and Cry2-mCherry-EB3, respectively, as compared to that of 130,000 A.U.– 380,000 A.U. for cells depicted in Fig. 1*B-D*). Right panels display magnified views of the regions within green squares in the left panels. Scale bar corresponds to 5 μm.

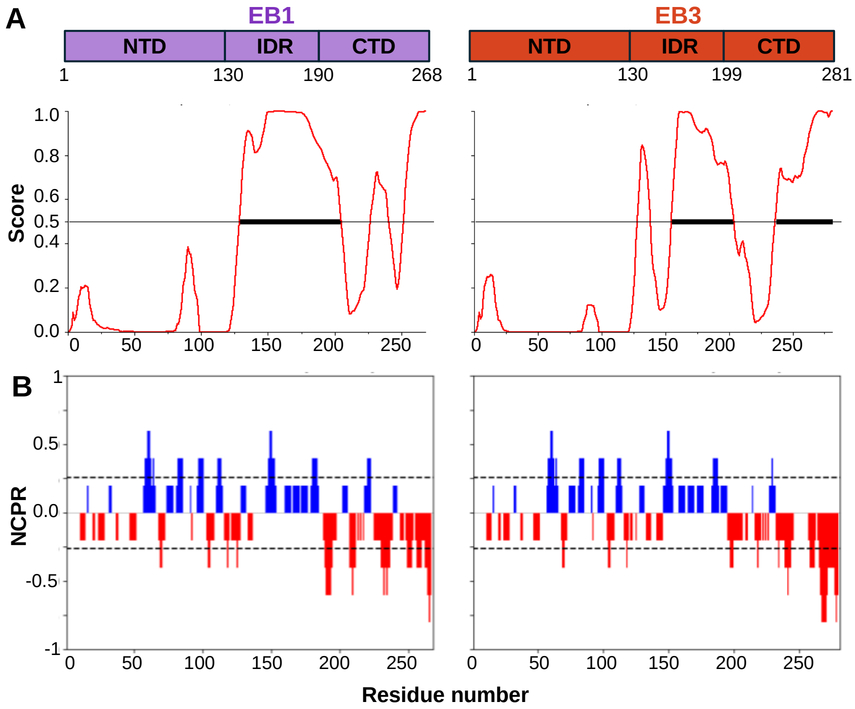

Figure S7. *A*, intrinsically disordered regions in EB1 and EB3 as predicted using the PONDR algorithm (53). Residues with scores above 0.5 are predicted to be disordered. *B*, net charge per residue (NCPR) plots for EB1 and EB2 showing polarized charge distribution in these proteins. The plots were generated using the algorithm available on CIDER (54) webserver with a 5-residue window.

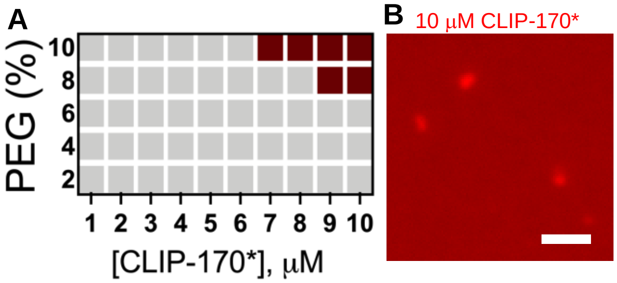

Figure S8. *A,* protein concentration versus PEG concentration phase diagram for CLIP-170*. Gray and brown boxes indicate the absence and presence of phase separation, respectively. *B*, representative fluorescence microscopy image of droplets formed by CLIP-170* (20 μM). CLIP-170* was labeled with Alexa Fluor 594 (red), and the ratio of labeled to unlabeled protein was 1:10. The image was obtained ~10 min after sample preparation; scale bar corresponds to 2 μm.

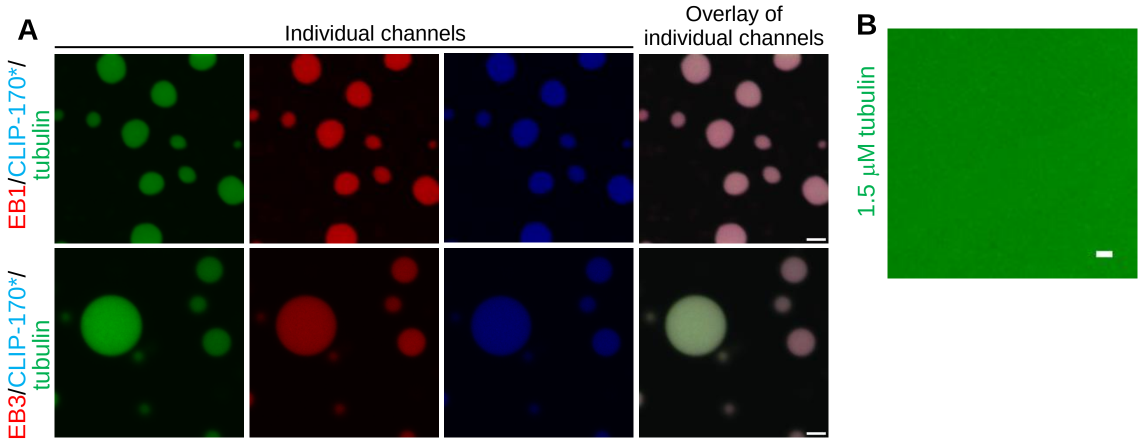

Figure S9. EB1 and EB3 form miscible droplets with CLIP-170* and tubulin. *A*, representative fluorescence microscopy images of EB1/CLIP-170* and EB3/CLIP-170* droplets in the presence of 0.5 µM tubulin. EB1 and EB3 (20 µM each) were labeled with Alexa Fluor 594 (red), CLIP-170* (10 µM) was labeled with Alexa Fluor 647 (far-red, changed to blue for better visualization), and the ratio of labeled to unlabeled protein was 1:10. Tubulin was labeled with HiLyte 488 (green). Scale bar corresponds to 5 µm. *B,* a representative fluorescence microscopy image of HiLyte 488-labeled tubulin (1.5 µM), showing that at this concentration tubulin alone does not assemble into microtubules under the present experimental conditions.

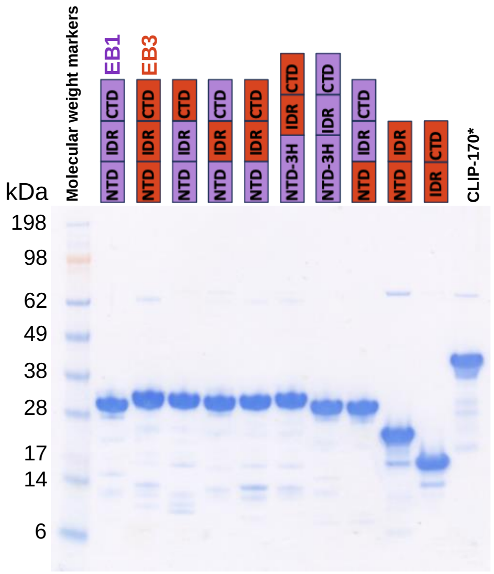

Figure S10. An image of the SDS-PAGE gel of purified proteins used in the present study. Protein purity was quantified by densitometry using the Fiji software. All proteins were >90% pure, except for ΔCTDEB3 (second last lane), which was ~85% pure.
